# Extracellular vesicles as an emerging mechanism of cell-to-cell communication

**DOI:** 10.1007/s12020-012-9839-0

**Published:** 2012-12-01

**Authors:** Ciro Tetta, Ezio Ghigo, Lorenzo Silengo, Maria Chiara Deregibus, Giovanni Camussi

**Affiliations:** Department of Molecular Biotechnology and Health Science and Department of Medical Sciences and Fresenius Medical Care, Translational Center for Regenerative Medicine, University of Torino, Via Nizza 52, Turin, Italy

**Keywords:** Exosomes, Microvesicles, MRNA, MicroRNA

## Abstract

The concept that extracellular vesicles may act as paracrine/endocrine effectors is based on the evidence that they are able to transport bioactive molecules between cells, either within a defined microenvironment or remotely, by entering the biologic fluids. Extracellular vesicles, including exosomes and microvesicles, may deliver lipids and various functional transcripts, released from the cell of origin, to target cells. Since extracellular vesicles contain defined patterns of mRNA, microRNA, long non-coding RNA, and occasionally genomic DNA, they may transfer genetic information which induces transient or persistent phenotypic changes in recipient cells. In this review, we will discuss potential physiologic and pathological implications of extracellular vesicles, as well as the diagnostic and therapeutic opportunities that they may provide.

## Introduction

Cells are required to communicate with each other for appropriate development and functioning of tissues. Classical means of cell communication are represented by cell junctions, adhesion contacts, and soluble factors that can act upon the same cell where they are produced, or upon neighboring cells, or may even act over long distances in an endocrine manner [1]. In addition to these described means, another mechanism of cell communication has recently emerged, namely communication by extracellular vesicles (EVs). EVs are released by numerous cell types such as blood cells, dendritic cells, endothelial and epithelial cells, nervous cells, tumor cells, and embryonic and adult stem cells in the extracellular space, both in physiologic and pathological conditions. EVs have also been identified in body fluids such as serum, saliva, amniotic fluid, synovial fluid, breast milk, and urine [2–7]. Cell communication by means of EVs is described as being a universal way for cells to interact with each other and influence the behavior of other cells by exchanging material and information.

EVs are cytosol fragments with spheroid morphology surrounded by a membrane composed of a lipid bilayer and hydrophilic proteins, similar to the cell plasma membrane. EVs are a heterogeneous group of vesicles, known in the literature by several different names (microvesicles, microparticles, ectosomes, exosomes, shedding vesicles, etc.), with sizes ranging from 30 to 1,000 nm. They are constitutively produced in vitro or in vivo by cells, or following the activation by soluble agonists or physical or chemical stress, including oxidative stress, hypoxia, and shear stress [8]. Since EVs carry receptors, bioactive lipids, proteins, and, most importantly, nucleic acids, such as mRNA and microRNA (miRNA), they are able to deliver important information to recipient cells. The delivery of mRNA to target cells is followed by subsequent transcription and production of functional proteins. Moreover, functional miRNA may interfere with the production of target proteins within recipient cells. Consequently, EVs may modify the phenotype and functions of target cells.

## Biogenesis of EVs

The exact process of EV formation is currently not fully elucidated. The previous classification of EVs into the two major groups of shedding vesicles and exosomes on the basis of their different biogenesis, size, and protein composition is presently controversial, and recent studies have questioned its validity.

It has been suggested that shedding vesicles may originate by direct budding from the cell plasma membrane into the extracellular space in a calcium-dependent process with cytoskeleton reorganization, curvature-mediated lateral redistribution of membrane components, leading to the creation of rafts and membrane nanodomains and formation of plasma-mediated attractive forces between membranes [9].

The instrumental role of sphingomyelinases (SMases) has been reported in the mechanism of EV release. It has recently been demonstrated that acidic-SMase (A-SMase) is involved in microparticle release in glial cells, and thus represents a crucial role in EV release. A-SMase activity triggers microparticle release from glial cells [10].

On the other hand, exosomes are thought to originate from the endosomal membrane cell compartment, and their release is said to be consequential to the exocytosis of multivesicular bodies and discharge into the extracellular space of intraluminal vesicles after fusion with the plasma membrane, in a p53-controlled process, and is dependent on cytoskeleton activation, but independent of cell calcium concentration. In addition, some studies have suggested that the multiprotein complex, Endosomal Sorting Complexes Required for Transport (ESCRT), has a critical role in the sorting of vesicles, and the finding of certain components of the ESCRT complex in exosomes, such as Alix and Tsg101, has raised the possibility that the ESCRT machinery could be involved in the formation and exocytosis of these vesicles [11, 12].

Trajkovich et al. [13] have recently suggested a different pathway for intra-endosomal membrane transport and exosome formation in a mouse oligodendroglial cell line, independent of the ESCRT machinery, but requiring sphingolipid ceramide. Ceramide is one of the major lipids in the lipid bilayer of cell membranes and is produced after hydrolysis of sphingomyelin, catalyzed by SMases, and used for the generation of intraluminal vesicles of multi-vesicular bodies that are not intended for transfer to lysosomes and subsequent degradation, but are released as exosomes [13].

Van Niel et al. [14] suggested that, in melanocytes, the CD63 tetraspanin accounts for an ESCRT- and ceramide-independent mechanism of EV sorting. In the biogenesis of melanosomes, this ESCRT-independent sorting regulated by CD63 tetraspanin could coexist with an ESCRT-dependent mechanism for predetermining the premelanosome protein, PMEL, for extracellular release via exosomes or for lysosome degradation [14].

A CD63 association has also been described for the viral oncogene latent membrane protein 1 (LMP1) in endosomes with low cholesterol concentrations that are released as exosomes to antagonize downstream NF-κB activation in Epstein Barr virus (EBV) infection of human B cells [15]. This study suggests an exploitation of distinct membrane subdomains by EBV-encoded LMP1, i.e., the aggregation of LMP1 in cholesterol-rich membranes of Golgi compartments to induce NF-κB activation, and the association of LMP1 with CD63 in microdomains, leading to the accumulation in intraluminal vesicles and consequent transfer, as exosomes, to outside the cell to prevent NF-κB activation.

Fang et al. [16] described a sorting pathway of exosomal proteins in Jurkat T cells, where protein selection was achieved on the basis of higher-order oligomerization and plasma membrane association, suggesting that this higher-order oligomerization was sufficient to target plasma membrane proteins to exosomes. Since higher-order oligomerization is a primary determinant of HIV Gag budding, they proposed a biogenesis similar to that of exosomes for HIV and other retroviruses [16].

## Composition of EVs

Many studies in the literature point to differential molecular expression by the two main classes of EVs, shedding vesicles and exosomes [17]. Shedding vesicles bear high concentrations of cholesterol and phosphatydilserine, along with molecules usually recognized in lipid rafts such as flotillin-1, but their molecular composition is highly heterogeneous, according to the various cells from which they originate. Tumor cells and neutrophils produce EVs enriched in proteolytic enzymes and metalloproteinases to degrade extracellular matrix. Platelets generate EVs carrying molecules, such as P-selectin, integrins, and GPIb and GPIIB-IIIa glycoproteins, critical for coagulation [17]. Conversely, exosomes show molecules on their surface, such as Alix, Tsg101, Hsc70, CD63, CD81, and CD9, thought to be distinctive of exosomes, as well as low amounts of phosphatydilserine.

However, the EVs produced by cells in the cell supernatant or present in extracellular fluids are inexorably a heterogeneous population of both shedding vesicles and exosomes, and it is currently very difficult to accurately discriminate between the two vesicle types.

## Functions of EVs

EVs can be internalized by recipient cells following receptor-ligand interactions and the varied assortment of bioactive molecules, derived from the cell of origin, such as proteins, bioactive lipids, and nucleic acids, can be transferred along with the proteins expressed on the EV surface.

EVs may directly activate the recipient cell by acting as signaling complexes [1, 17]. In fact, EVs derived from macrophages bind to platelets by means of the P-selectin glycoprotein ligand-1 expressed on their surface and EVs from neutrophils expressing Mac-1 may induce platelet activation.

EVs may also transfer receptors from one cell to another. Quah et al. [18] have demonstrated that bystander B cells can rapidly acquire antigen receptors from activated B cells by membrane transfer with the resulting increase of a cell population presenting a specific antigen to CD4 T cells. Also, Fas ligand can be transferred from tumor cells by EVs provoking activated T cell apoptosis [19]. In addition, tumor cells may transfer Tissue Factor (TF) and oncogenic receptors such as EGFRvIII to neighboring endothelial cells, via EVs, thus potentiating tumor angiogenesis [20, 21].

Moreover, EVs may convey proteins to the cytoplasm of recipient cells, such as the cell death caspase-1 message conveyed by microvesicles derived from LPS-stimulated monocytes [22], or the tumor exosome-carried Notch ligand Delta-like 4 which inhibits Notch signaling, enhancing angiogenesis [23].

Recent studies have also reported the release of EVs from neurons to glial cells, as well as their presence in cerebrospinal fluid [24, 25]. Al-Nedawi et al. [26] demonstrated that glioblastoma multiforme tumor cells expressing the truncated and oncogenic variant of the epidermal growth factor receptor can transfer this receptor to other cells, via microvesicles, in this way propagating the oncogenic phenotype. In the central nervous system, it has been suggested that some proteins such as beta-amyloid peptide, prion protein, tau, and alfa-synuclein are transferred via exosomes, thus avoiding degradation and favoring the deposition of pathogenic aggregates in neurodegenerative diseases (Alzheimer Disease, Tauopathies, prion disease, and Parkinson Disease) [27]. In addition, exosomes may contribute to the diffusion of prions [28].

The most interesting aspect of EV function is their involvement in the transfer of genetic information. In addition to bioactive proteins and lipids, EVs also contain nucleic acids such mRNA and miRNA, thus protecting them from extracellular degrading enzymes. In particular, the horizontal transfer of miRNAs has been proposed as a new form of intercellular communication, representing a means by which donor cells can regulate gene expression of recipient cells [29]. In fact, miRNAs are 19–23 nucleotide-long non-coding RNAs known as critical post-transcriptional modulators of gene expression. Present both in plants and metazoans, miRNAs are able to induce inhibition or degradation of target mRNAs, thus influencing many processes of cell homeostasis, such as survival, proliferation, and cell differentiation, as well as metabolism and tumorigenesis processes [30]. In mammals, miRNAs play the role of finely regulating and controlling about half of their mRNAs. Hundreds of mRNAs can be targeted by a single miRNA, and one mRNA can be repressed by different miRNAs in a complex interrelated network. In the nucleus, one particular RNase III family member, Drosha, converts primary miRNA transcripts into 70-nucleotide-long pre-miRNA [30]. Exported from the nucleus into the cytoplasm by exportin 5, the pre-miRNA is then cleaved by the RNase III Dicer to generate a mature miRNA duplex, approximately 22-nucleotides long. Some miRNAs are created by a process of splicing and debranching, bypassing the Drosha step in the nucleus, but still involving cleavage by Dicer in the cytoplasm. In the following steps, the guide strand of the miRNA duplex is integrated into the miRNA-induced silencing complex (miRISC), while the passenger strand is degraded. Argonaute 2 (Ago2) protein is a core component of miRISC and directly cooperates with miRNAs in the repression of target mRNAs [30]. The cytoplasmatic foci known as processing bodies and stress granules are dynamic structures where the translationally repressed mRNAs can be accumulated while waiting to be recycled or degraded. Both of these foci essentially share some components of the miRISC complex [30]. Interestingly, EVs contain components of the miRISC complex, such as Ago2, together with several RNA-binding proteins known to regulate RNA traffic between the nucleus and the cytoplasm. It can be therefore hypothesized that, during EV biogenesis, these RNA binding proteins regulate the accumulation of selected RNAs within EVs. Studies on the transfer of reporter mRNAs and their translation into proteins, demonstrated both in vitro and in vivo, suggest that the mRNA delivered by EVs is functional [31–33]. The presence of miRNAs inside exosomes derived from mouse and human mast cells was shown by Valadi et al. [34]. In addition, Yuan et al.^.^ [35] demonstrated the in vitro transfer of miRNAs to murine embryonic fibroblasts by EVs. We have characterized miRNAs critical for cell survival and differentiation, as well as multi-organ development and immune system regulation in EVs from human mesenchymal stem cells. These miRNAs can be transferred by EVs to recipient cells and can modulate the expression of target proteins [36, 37].

Recent studies suggest that EVs can also contain DNA. Waldenström et al. [38] showed that, in addition to mRNA, EVs derived from cultured adult murine cardiomyocytes can also transfer chromosomal DNA to fibroblasts, confirming the previous data demonstrating the occurrence of DNA in human prostasomes [39]. Guescini et al. [40] also reported that exosomes derived from astrocytes and glioblastoma cells can carry mitochondrial DNA.

## Effect of EVs in immune response, tumor, and stem cell biology

The miRNAs expressed by cells of the immune system may influence pathways controlling the development and role of innate and adaptive immune responses, and they may be dysregulated in cancers, thus acting as tumor suppressors or oncogenes [41]. The findings that Epstein-Barr virus-transformed B lymphocytes produced exosomes expressing MHC class II dimmers, and were able to present them to T cells [42], and that dendritic cell-derived exosomes expressing MHC class I–peptide complexes can induce CD8+T-lymphocyte-dependent antitumor immune responses in mice in vivo [43] both suggest that exosomes could be critical modulators of the adaptive immune response. The message delivered by exosomes differs depending on the physiologic state of the cell from which they derive [44, 45]. Only recently it has been suggested that miRNAs delivered by EVs could play a role in immune system regulation [46]. In fact, an EV-dependent exchange of miRNAs between APC and T cells occurs at the site of immune synapses [47]. It has been shown that EVs are directly involved in the cognate DC–T cell interaction [48]. These studies suggest the involvement of a dynamic exchange of information between DCs and T cells through EVs [46].

EVs have been also implicated in the modulation of the tumor microenvironment. Indeed, tumor-derived EVs may inhibit the immune response, thus favoring the tumor cell escape from immune surveillance [49] by inducing apoptosis in immune effectors such as NK cells and cytotoxic T lymphocytes (CTLs). In addition, EVs derived from human prostate cancer cells transport the Fas ligand, which may be transferred to CTLs, favoring their apoptosis [50].

Moreover, tumor-derived EVs may induce phenotypic changes in stromal cells that favor tumor invasion and dissemination [51]. Specifically, EVs may promote the exchange of receptors, active proteins, lipids, or genetic information between tumor and stromal cells; for example, tumor-derived EVs may enhance tumor invasion by delivering interleukin-8 and chemokines [52] or matrix metalloproteinases (MMP) and extracellular MMP inducer [51] to neighboring cells. The horizontal propagation, via EVs, of the oncogenic form of epidermal growth factor receptor EGFRvIII from highly aggressive to non-aggressive glioma cells may promote tumor growth and invasion [26]. Several other transcripts capable of favoring tumor invasion and metastasis were shown to be transferred from cancer to stromal cells, such as transglutaminase and fibronectin [53] and Delta-like ligand 4 [23].

In addition, EVs may transfer genetic information (mRNA, miRNAs, and oncogenes) from tumor cells, capable of reprogramming normal stromal and endothelial cells. The phenotype of tumor-associated monocytes has been shown to depend on the transfer of tumor mRNA [54].

As shown by Skog et al. [55], glioblastoma-derived EVs may transfer specific and functional mRNA and miRNAs capable of inducing activation of cell migration, angiogenesis, and proliferation in brain microvascular cells. EVs derived from other tumors such as colorectal[56], lung [57], and prostate cancer cells [58] alter the phenotype of normal cells by transferring specific RNA subsets. On the other hand, EVs released from the surrounding cells may modify cancer cell gene expression [58]. EVs derived from cancer stem cells were shown to contain pro-angiogenic RNAs able to induce a premetastatic niche in the lungs, whereas those derived from differentiated cancer cells were not able to induce this niche, and their mRNA and miRNA content differs [59]. EVs from cancer stem cells contained miR-29a, miR-650, and miR-151, all associated with tumor invasion and metastases, along with miR-19b, miR-29c, and miR-151, known to be upregulated in patients with renal carcinomas [59].

Another emerging field on the role of EVs is in stem cell biology. Studies by Ratajczak et al. [60] demonstrated that EVs derived from embryonic stem cells are involved in the maintenance of pluripotency and the undifferentiated phenotype of stem cells. It has been subsequently shown that EVs from embryonic stem cells are enriched in miRNAs which can be transferred in vitro to murine embryonic fibroblasts [35]. Adult stem/progenitor cells may also exploit EVs to convey genetic information. We found that EVs released from endothelial progenitor cells may transfer a specific subset of mRNA, associated with angiogenic pathways, to quiescent endothelial cells, capable of activating an angiogenic program in these cells [31]. Subsequent studies based on silencing of Dicer to generate miRNA-depleted EVs, or on selective inhibition of angio-miRNAs (miR 126 and miR 296) demonstrated that the angiogenic effect of EVs released by endothelial progenitor cells are mainly dependent on miRNA transfer [61]. Quesenberry et al. [62] have suggested a continuum model of stem cell biology in which EVs convey information determinant for stem cell differentiation. According to this theory, EVs, in concert with the cell cycle transit of stem cells, are critical factors for modulation of stem cell plasticity.

## EVs as a diagnostic tool

Based on the evidence that EVs are present in blood and other biologic body fluids, EVs can be regarded as potential easily accessible diagnostic biomarkers for many pathological conditions, including metabolic diseases such as type II diabetes and obesity [63]. Patients with type II diabetes show an increased amount of platelet- and monocyte-derived EVs [64]. In particular, the number of EVs is elevated in diabetic nephropathy [65, 66]. An elevated number of platelet-released EVs have also been reported in diabetic patients after acute myocardial infarction, compared with non-diabetic subjects with myocardial infarction [67]. The urinary level of EV-associated dipeptidyl peptidase-IV in type II diabetic patients was found to be higher than in normal subjects, suggesting that these EVs could be exploited as specific biomarkers for the onset of diabetic nephropathy [68]. Muller et al. [69] proposed that insufficient lipid accumulation and lipid droplet biogenesis with defective conversion from small to large adipocytes in obesity is related to a defect of EV-mediated communication.

Several studies have addressed the potential use of EVs as biomarkers in cancer as their level in circulation correlates with a poor prognosis. Mucin-expressing EVs are potential markers for early detection of adenocarcinomas [70]. Moreover, patients with melanoma have high levels of exosomes expressing CD63 and caveolin-1 in plasma, as detected by sandwich ELISA [71].

Circulating EVs were shown to contain tumor-specific mRNA in glioblastoma [55], as well as in gastric [72] and breast [73] carcinomas. In particular, the miRNA profile of EVs can be useful for diagnostic purposes in cancer [74]. For example, the profile of miRNA may vary with the disease stage in ovarian cancer [75], and may have a diagnostic potential in lung adenocarcinoma [76].

EVs present in urine could be also used as a biomarker. EVs in urine of patients with prostate cancer express mRNA encoding PCA3 and the TMPRSS2:ERG fusion products which are up-regulated in cancer cells [7]. Several diagnostic products based on EVs/exosomes have recently been developed by commercial companies [77]. Exosome Sciences has developed an ELISA test for identification of exosomes in HIV, tuberculosis, and cancer (www.AethlonMedical.com). The Exotest based on an ELISA platform is under development for neurodegenerative and cancer diseases by HansaBioMed (www.HansaBioMed.eu). Exosome Diagnostic has created an EV-based platform, mainly for cancer diagnostic purposes (www.ExosomeDX.com). Caris Life Sciences has focused on a sensitive EV-based diagnostic test for prostate cancer (www.CarisLifeSciences.com). In summary, the use of EVs as a diagnostic tool may provide an easy and non-invasive way to detect tissue-derived markers specific for the cell of origin.

## Therapeutic potential of stem cell-derived EVs

The concept that EVs can modulate the phenotype of target cells by transferring extracellular RNAs is an emerging paradigm in intercellular communication. According to this view, extracellular RNAs are now considered as paracrine/endocrine signals. EVs contain diverse species of RNAs including mRNA, miRNAs, and long non-coding RNAs that reflect the functional state of the cell of origin. Therefore, several studies have evaluated whether stem cell-derived EVs may mimic stem cell functions. Recent studies on adult stem cell-induced tissue regeneration point to paracrine/endocrine mechanisms rather than permanent engraftment and trans-differentiation of stem cells. Therefore, the paracrine/endocrine hypothesis has changed the perspective of the therapeutic use of stem cells in regenerative medicine. Several studies indicate that EVs play a relevant role as mediators of stem cell-induced regeneration by reprogramming injured cells.

In experimental acute kidney injury, EVs derived from MSCs were shown to mimic the beneficial effects of stem cell treatment by reprogramming injured tubular epithelial cells to a stem cell-like phenotype by triggering a regenerative program [78]. While EV-transferred mRNA can be translated into proteins, the miRNAs are post-transcriptional regulators which may induce epigenetic changes in recipient cells. In these experimental conditions, EVs were shown to accumulate at the site of tissue injury and to transfer MSC-specific functional RNAs including regulators of transcription capable of altering gene expression in recipient cells, such as upregulation of BCL-XL, BCL2, and BIRC8 anti-apoptotic genes, and downregulation of CASP1, CASP8, and LTA genes involved in cell apoptosis [79]. The renoprotective action of EVs derived from endothelial progenitor cells in renal acute ischemia reperfusion injury was mainly ascribed to the transfer of pro-angiogenic miRNAs [61]. EVs derived from endothelial progenitor cells were also found to improve neovascularization and muscle regeneration in a model of hind limb ischemia [80]. In pig and mouse models of acute infarction, EVs were shown to limit the infarct size and favor recovery [81].

**Fig. 1:**
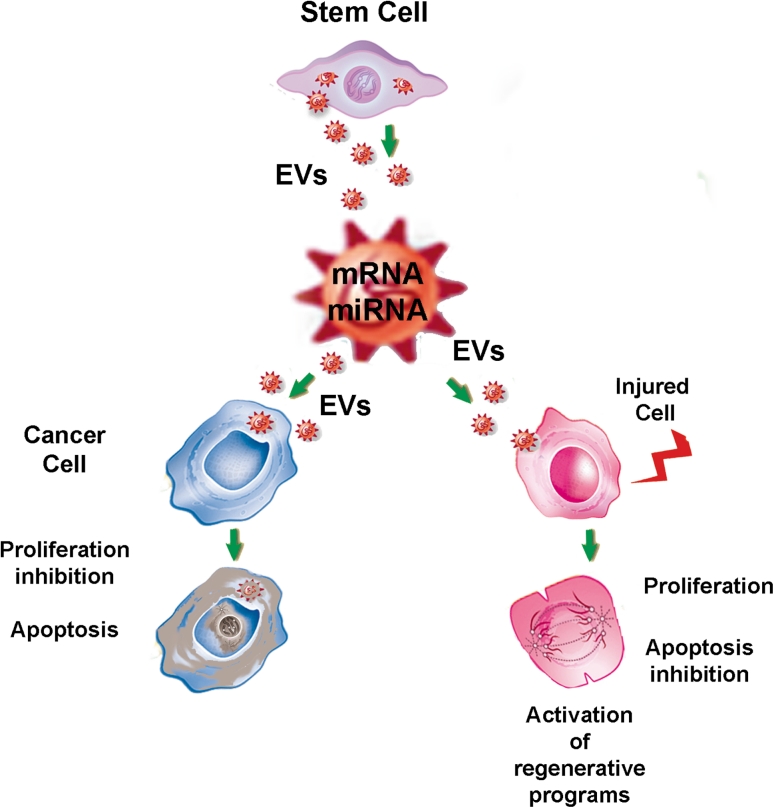
Differential effects of stem cell-derived EVs. The effects of EVs not only depend on their content but also on which pathways are activated in the recipient cells. In particular, the miRNA contents of EVs have different effects depending on the state of activation of their targets. The same EVs released from stem cells may stimulate activation of regenerative programs in injured cells leading to their dedifferentiation, cell cycle re-entry with proliferation, and promoting cell survival. Conversely, the same EVs may reprogram cancer cells by delivering tumor suppressive miRNAs which inhibit tumor cell growth and promote apoptosis

The biologic effects of EVs, however, not only depend on their content but also on the metabolic and functional state of recipient cells. In fact, the delivery of miRNAs may have differential effects on injured, normal, or neoplastic cells depending on which pathways are activated in the recipient cells (Fig. 1). For instance, EVs derived from human liver stem cells may activate regenerative programs in hepatocytes, favoring liver regeneration after 70 % hepatectomy in rats [82]. Conversely, the same EVs may reprogram HepG2 hepatoma and primary hepatocellular carcinoma cells (HCC) by inhibiting their growth and survival, leading to regression of ectopic tumors developed in SCID mice [37]. The role of tumor-suppressive miRNAs delivered by EVs was suggested by evidence of their active transfer to tumor cells, and the abrogation of the biologic activity of EVs with depleted miRNA [37]. Studies aimed at defining the molecular components of EVs responsible for their biologic actions in different pathological conditions, and understanding the mechanisms involved in specific packaging of RNAs could subsequently lead to developing strategies for engineering the contents of EVs for therapeutic purposes.

## Conclusions

EVs are able to alter the behavior of recipient cells by transferring bioactive molecules to them. EVs released by a given cell type may function within a defined microenvironment or may even act at a distance. The exchange of information between cells by EVs is frequently bidirectional. As EVs contain molecules which are characteristic of the cell of origin, they may be exploited as biomarkers. In addition, their ability to deliver genetic material from stem to tissue-injured cells can, at least partly, explain the paracrine/endocrine action of stem cells. Pre-clinical studies suggest that EVs can reproduce the effect of stem cell treatment, prompting research to evaluate whether they can be a substitute for cell-based therapy in regenerative medicine.
